# Using Wearable Sensors and Machine Learning to Assess Upper Limb Function in Huntington’s Disease

**DOI:** 10.21203/rs.3.rs-4355136/v1

**Published:** 2024-06-03

**Authors:** Adonay S. Nunes, İlkay Yıldız Potter, Ram Kinker Mishra, Jose Casado, Nima Dana, Andrew Geronimo, Christopher G. Tarolli, Ruth B. Schneider, E. Ray Dorsey, Jamie L. Adams, Ashkan Vaziri

**Affiliations:** 1. BioSensics LLC, 57 Chapel St, Newton, MA, USA; 2. Penn State College of Medicine, Hershey, PA, USA; 3. Center for Health + Technology, University of Rochester Medical Center, Rochester, NY, USA; 4. Department of Neurology, University of Rochester Medical Center, Rochester, NY, USA

**Keywords:** Huntington’s disease, upper limb function, wearable sensors, accelerometer, digital health biomarkers

## Abstract

Huntington’s disease (HD), like many other neurological disorders, affects both lower and upper limb function that is typically assessed in the clinic - providing a snapshot of disease symptoms. Wearable sensors enable the collection of real-world data that can complement such clinical assessments and provide a more comprehensive insight into disease symptoms. In this context, almost all studies are focused on assessing lower limb function via monitoring of gait, physical activity and ambulation.

In this study, we monitor upper limb function during activities of daily living in individuals with HD (n = 16), prodromal HD (pHD, n = 7), and controls (CTR, n = 16) using a wrist-worn wearable sensor, called PAMSys ULM, over seven days. The participants were highly compliant in wearing the sensor with an average daily compliance of 99% (100% for HD, 98% for pHD, and 99% for CTR). Goal-directed movements (GDM) of the hand were detected using a deep learning model, and kinematic features of each GDM were estimated. The collected data was used to predict disease groups (i.e., HD, pHD, and CTR) and clinical scores using a combination of statistical and machine learning-based models.

Significant differences in GDM features were observed between the groups. HD participants performed fewer GDMs with long duration (> 7.5 seconds) compared to CTR (p-val = 0.021, d = −0.86). In velocity and acceleration metrics, the highest effect size feature was the entropy of the velocity zero-crossing length segments (HD vs CTR p-val <0.001, d = −1.67; HD vs pHD p-val = 0.043, d=−0.98; CTR vs pHD p-val = 0.046, d=0.96). In addition, this same variable showed a strongest correlation with clinical scores. Classification models achieved good performance in distinguishing HD, pHD and CTR individuals with a balanced accuracy of 67% and a 0.72 recall for the HD group, while regression models accurately predicted clinical scores. Notably the explained variance for the upper extremity function subdomain scale of Unified Huntington’s Disease Rating Scale (UHDRS) was the highest, with the model capturing 60% of the variance. Our findings suggest the potential of wearables and machine learning for early identification of phenoconversion, remote monitoring in HD, and evaluating new treatments efficacy in clinical trials and medicine.

## 1 Introduction

Huntington’s disease (HD) poses significant challenges due to its complex motor, cognitive, and behavioral symptoms. HD is an inherited autosomal dominant neurodegenerative disorder that manifests in midlife and progresses steadily, affecting individuals’ motor functions, cognition, and behavior (Walker, 2007). Particularly intriguing is the period preceding clinical diagnosis, known as prodromal HD (pHD), during which symptoms may emerge, offering a window for early intervention (Papp et al., 2011). However, the lack of disease-modifying therapies underscores the urgency of accurate and timely monitoring to facilitate early intervention. Currently, the Unified Huntington’s Disease Rating Scale (UHDRS) is the primary tool used for assessing motor function, cognitive abilities, and behavioral symptoms in HD. It provides a comprehensive overview of a patienťs functional capabilities and disease progression. While UHDRS provides critical snapshots of a patienťs condition at specific points in time, wearable technology can supplement these by offering continuous, objective, and personalized data, thereby enhancing the monitoring and management of HD.

In this context, frequent at-home monitoring emerges as a critical tool for tracking disease progression and assessing treatment efficacy. Automated remote monitoring offers several advantages over traditional clinic-based assessments, including increased frequency, reduced subjectivity, and the ability to capture subtle changes in motor function (Andrzejewski et al., 2016; Bennasar et al., 2018; Dorsey et al., 2017; Ó Breasail et al., 2021; Sharma et al., 2023). In addition, remote monitoring technologies have the potential to reduce the burden of clinical care and research by moving assessments from the clinic to the home, potentially expanding access for diverse patient populations. In this context, the use of wearable sensors can provide a sensitive tool for tracking upper limb function during activities of daily living (Bennasar et al., 2016; Tang et al., 2020; Troiano et al., 2014; Zhou et al., 2008). Specifically, goal-directed movements (GDMs), which are fundamental to daily activities like reaching and grasping, serve as atomic components of upper limb movements and offer valuable insights into motor function (Desmurget et al., 1998; Elliott et al., 2010).

Recent advancements in deep learning have propelled the development of robust techniques for automated GDM detection from accelerometer data (Elkholy et al., 2020; McLeod et al., 2016; Panwar et al., 2019; Subash et al., 2022). Leveraging these advancements, we have developed PAMSys ULM (ULM: upper limb monitoring; BioSensics LLC, Newton, MA), a wearable sensor for continuous monitoring of features of GDMs during activities of daily living (A. S. Nunes et al., 2023). PAMSys ULM has been used for monitoring upper limb (UL) function in several neurodegenerative conditions including stroke (A. S. Nunes et al., 2023), Friedrich’s ataxia (R. Mishra et al., 2024), and ALS (A. S. Nunes et al., 2024), as well as a recent study in inclusion body myositis (R. K. Mishra et al., 2024). This study aims to validate the effectiveness of the PAMSys ULM in assessing upper limb function in HD by examining the correlation between sensor-derived features and established clinical scores. We postulated that GDM features would be able to identify group differences between individuals with HD, pHD, and healthy controls (CTR), and that these differences would be correlated with the clinical scores. In addition, we used machine learning-based models to classify the groups based on the GDM features and predict their clinical scores.

## 2 Methods

### 2.1 Experimental Design

The experimental setup was previously reported in (A. Nunes et al., 2024), where speech data were used. In brief, participants provided written informed consent and were enrolled in an investigator-initiated observational cohort study performed at the University of Rochester. The study was reviewed and approved by the University of Rochester Institutional Review Board. The longitudinal study included visits every three to 6 months, for up to 3 years of total follow-up. During each visit, HD, and pHD participants were assessed via the UHDRS (Kieburtz et al., 1996) and their demographics, concomitant medications, and health history were collected. To derive a clinical assessment of the upper limb function, we use the upper extremity function subdomain scale of UHDRS (UHDRS-UL). UHDRS-UL is calculated by adding the following UHDRS item scores for both upper extremities: finger tapping, pronate/supinate, rigidity of the arms, maximal dystonia, and maximal chorea of the upper limbs. Following the visit, all participants wore a wrist sensor on the dominant hand and a pendant sensor on the chest for one week, with instructions to wear the sensors continuously. This study uses solely the wrist sensor accelerometer data. Similarly, for BioDigit Database CTR participants, the Penn State University Review Board approved the study where participants wore a wrist sensor for a week.

### 2.2 Participants

16 individuals with HD, 7 individuals with pHD, and 11 CTR were included in the study. HD status was confirmed clinically by a movement disorders specialist investigator and either a self-reported first degree relative with HD or self-reported genetic test indicating a CAG expansion of >36 in the *huntingtin* gene (Walker 2007). Prodromal HD participants were individuals with a self-reported CAG expansion of >36 in the *huntingtin* gene (Walker 2007) without a clinical diagnosis of HD. CTR participants were individuals in good health with no evidence of neurological disorders likely to cause involuntary movements or gait disturbance, as determined by the investigator. They were age-matched to the participants in the HD group. Age and sex were not significantly different between HD and CTR groups, but pHD had significant differences for both groups. Exclusion criteria included pregnancy and any neurological, medical, or psychiatric conditions that would preclude participation in the activities in the investigator’s judgment. In addition, we used data from 5 sex- and age-matched healthy CTR who underwent the same type of monitoring for a week from BioDigit Database, a database of digital health data created and maintained by BioSensics (Table 1).

### 2.3 Data analysis

Raw accelerometer data was preprocessed as described by Nunes et al. 2024. In brief, the data was first bandpass filtered between 0.1 and 12 Hz with a 4th order Butterworth filter to remove the inertial gravity component and high frequency activity, and then down sampled to 25 Hz. The velocity was estimated by integrating the acceleration data. A deep learning model was used to detect 3-second windows with a minimum 1.5 seconds of GDM. For each window, a total of 8 features for acceleration and for velocity magnitudes were calculated, including minimum, maximum, and median features. For zero-crossing features, three-axis components were used to calculate zero-crossing count, duration, and duration entropy. Zero crossing features in acceleration and velocity analysis detect shifts in movement direction or speed by counting shifts from positive to negative (or vice versa) and measuring the duration and variability of these shifts. These features are important as they can capture chorea, tremor movement or overshooting (Keenan & Wilhelm, 2005; Klapper et al., 2003). In addition, for each recording, the total count and count per GDM duration were calculated. The features were grouped per day and the median was extracted, then the mean across days was calculated. The median was used to avoid the influence of any possible outlier.

Statistical significance between groups was calculated with t-tests, and false discovery rate (FDR) correction was applied to identify group differences that survived multiple comparisons. Spearman correlations were used to assess the association between GDM features and the clinical assessments, as clinical scores were reported on an ordinal scale.

Machine learning was used to classify individuals in HD, pHD, and CTR groups, and to predict the clinical scores of UHDRS functional, UHDRS motor, total functional capacity, and UHDRS-UL. Maximal correlation feature selection was used to select the top 5 features as input for the models. An elasticnet regression model was trained for regularization, and a logistic layer was added for classification. Leave-one-subject-out cross validation was used to test model performance. In some instances a few subjects had one extra visit, thus, the total number of data points for classification was HD: 18, pPH: 8, CTR: 21. For regression, the total number of data points with available clinical scores was HD: 17, pPH: 8, CTR: 14. In the regression model and correlation, all the groups were included to capture all the health spectrum, ensuring that models performance are tested from high severity to healthy individuals. Classification performance was tested using balanced accuracy and recall, and regression prediction performance with mean squared error, mean absolute error, correlation score, and explained variance.

## 3 Results

The participants were highly compliant in wearing the PAMSys ULM wrist sensor with an average daily compliance of 99% (100% for HD, 98% for pHD, and 99% for CTR) - In total, two participants (one pHD and on CTR) did not wear the sensor for 1 days during the 7 day monitoring period.

**Figure 1.**
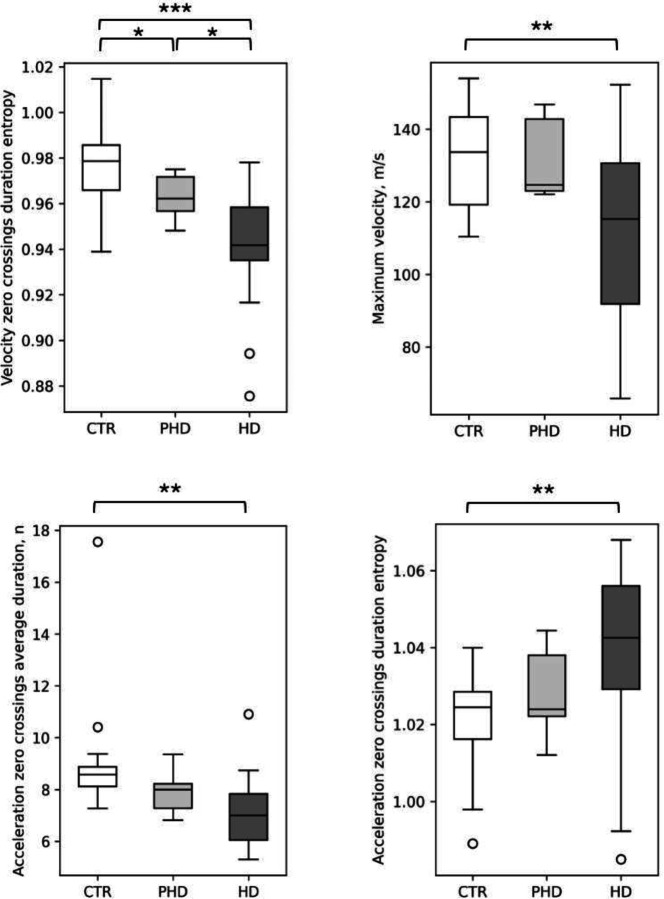
Scatterplot of GDM features across groups. The selected features were significantly different between HD and CTR participants. Although pHDs were not significantly different in the majority of features, it can be noted that the pHD mean lies in between the CTR and HD levels. * indicate significance <0.05, ** <0.01, *** <0.001

Features extracted from GDM periods and averaged per participant were compared between groups. Several features were significantly different as presented in Table 2, with selected features shown in Figure 1. Individuals with HD performed significantly fewer GDM movements with long duration (> 7.5 seconds) compared to the CTR group (p-val = 0.021, d = −0.86). Notable distinctions emerged in velocity-related features during GDMs between HD and CTR participants. Specifically, median velocity (p-val = 0.019, Cohen’s d = −0.89), maximum velocity (p-val = 0.01, d = −0. 89), and velocity root mean squared (p-val = 0.015, d = −0.94) were greater in CTR participants. Acceleration features that significantly differed between HD and CTR were zero-crossing related, namely, the average number of zero-crossings (p-val = 0.018, d = 0. 92), the entropy of the zero-crossing length (p-val = 0.01, d = 0. 98) and zero-crossing average duration length (p-val = 0.012, d = −0. 99). This indicates that HD individuals have more jerky movements, on average with shorter duration, while the distribution of the GDMs duration is more scattered. As seen in Figure 1, features have a trend with CTR and HD mean values at opposite ends and pHD mean values in between the two groups. However, with the current pHD sample size the differences are not significant despite having similar effect sizes.

**Figure 2.**
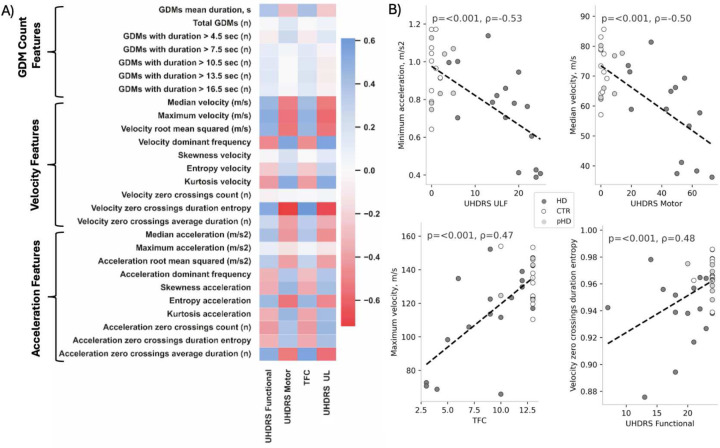
Correlations between GDM features and clinical assessment scores. A) heatmap showing all the correlations. It can be noted that velocity and acceleration features correlate the most with the clinical scores. B) Scatterplots for each clinical assessment and a GDM feature which was significantly correlated.

Correlations between count-based features and clinical scores were not significant. Median, maximum, root mean squared, and zero-crossing duration entropy velocity features correlated significantly with all the clinical scores with negative correlation values ranging from −0.71 to 0.59. Acceleration features that significantly correlated with all the clinical scores were entropy and zero-crossing average duration, with correlation values ranging from −0.52 to 0.51. All the correlations are illustrated in Figure 2A as a heatmap, and selected features plotted as a scatterplot in Figure 2B. The correlation values and significance are provided in the supplementary materials (Table S1)

**Figure 3.**
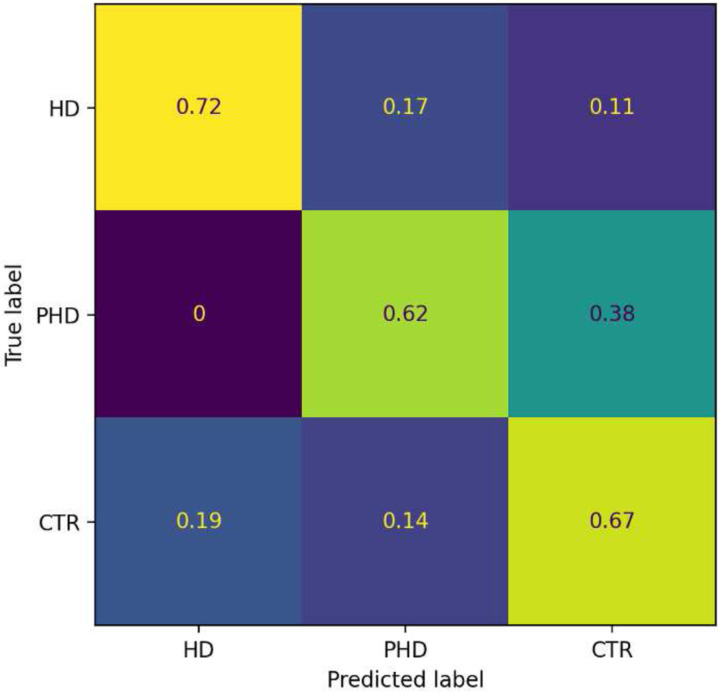
Confusion matrix for group classification. The percentage of subjects is presented in each box. The diagonal values indicate the recall, the percentage of group subjects correctly identified. Total data points per group was 18 HD, 8 pPH and 21 CTR.

A logistic regression with an elasticnet regularization was used to classify individuals in HD, pHD and CTR groups. Balanced accuracy was used as the main metric of performance and the model achieved a balanced accuracy of 0.67, with 0.33 being the chance level. The recall for the HD group was 0.72, for the pHD 0.62 and for the CTR 0.67. Figure 3 shows the corresponding confusion matrix. It can be noted that the model had more difficulties in distinguishing pHD from controls, due to the small differences between them.

**Figure 4.**
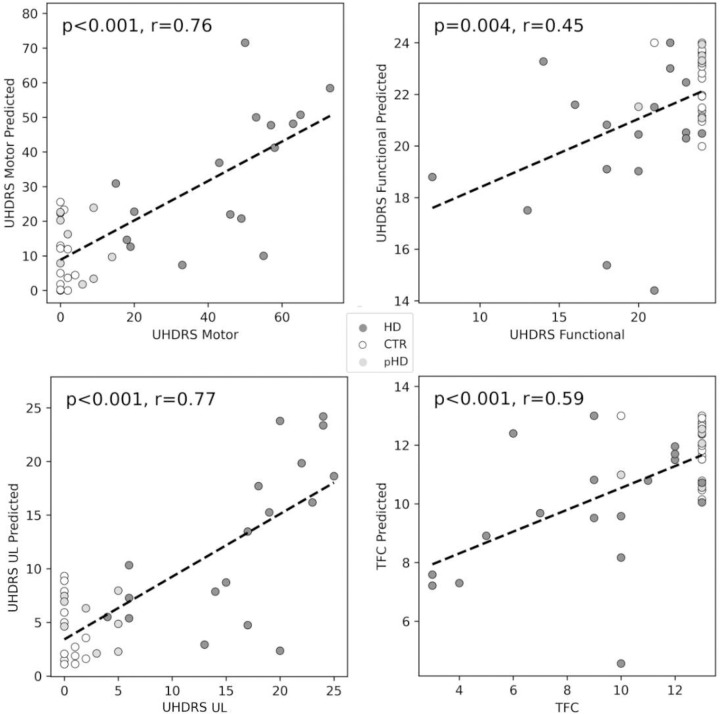
Clinician-rated clinical scores vs. predicted scores scatterplots. The predicted scores were from the leave one out test data. *TFC: Total functional capacity, UL: upper limb*

For predicting clinical scores, regression models with elasticnet regularization were used. Table 3 shows the models’ performance. The highest explained variance was achieved with the UHDRS UL explaining 60%, showing that GDM features are good candidates for predicting upper limb function. UHDRS motor scores were also predicted with good explained variance with 56% of the variance captured by the model. Total Functional Capacity performance was moderate with 33% of the explained variance captured by the model. Figure 4 shows the scatterplots of the actual and predicted scores.

## 4 Discussion

The study presents a novel approach to monitoring upper limb function in individuals with HD and pHD using accelerometer data collected over a span of seven days. This method offers several advantages, including the ability to potentially provide more precise and frequent assessments in a natural living condition and capturing subtle changes in motor function that may not be evident during clinic-based evaluations. Our approach encompassed several key steps. Firstly, we examined group differences and correlations in goal-directed movements (GDMs) between individuals with HD, prodromal HD, and CTR participants. Subsequently, leveraging machine learning techniques, we trained models to gauge the informative nature of GDM features for two main purposes: classifying individuals into HD, prodromal HD and CTR groups, and predicting clinical scores, including the UHDRS UL score. Through this multifaceted approach, we aimed to elucidate the potential of accelerometer data for remote monitoring and early intervention strategies in HD, specifically, how automated GDM detection can be used to monitor upper limb function in HD.

Results from this study indicate significant differences in the number of GDM counts, velocity-related GDM features between individuals with HD and CTR participants. Notably, HD participants demonstrated fewer and shorter-duration GDMs, which could be due to increased pauses during movements, potentially in the setting of emergent competing motor features (e.g., chorea). In addition, HD participants had lower median velocity, maximum velocity, and velocity root mean squared values than CTR. Similarly, acceleration-related features, such as zero-crossing metrics, differed significantly between HD and CTR groups, suggesting differences in movement characteristics between the two cohorts. While decreased velocity indicates GDM movements that are performed slower, zero-crossing features indicate jerkiness in the movements, being less smooth with a zigzag pattern where acceleration changes signs. These results are in accordance with previous studies that have shown upper limb difficulties in movement control characterized by target overshooting and movement overcorrections when performing goal-oriented movements (Gordon et al., 2019; Klein et al., 2011; Lemay et al., 2008), in addition to involuntary choreatic movements (Mann et al., 2012; Reilmann et al., 2011)

Classification models utilizing machine learning techniques showed promising results in classifying individuals into HD, prodromal HD, and CTR groups, with good performance particularly in distinguishing the HD group with respect to several GDM-based features. To potentially aid early identification, zero crossing entropy of velocity in particular exhibited significant difference between pHD and control groups, as this feature is governed by both the rate of sign changes and uncertainty in the measured velocity. Moreover, regression models demonstrated the ability to predict clinical scores with significant correlations, with the UHDRS UL score showing the highest explained variance. This suggests that GDM features extracted from accelerometer data could serve as valuable predictors of upper limb function, providing insights for experts to monitor disease progression and treatment efficacy.

However, the study has some limitations, notably the small sample size, particularly in the prodromal HD group, which may limit the generalizability of the findings. Effects sizes in pHD were notable but due to the sample size significance was not reached. In addition, age and sex differences in the pHD groups compared to the other groups might further hinder finding significantly different features. Future studies with larger sample sizes, especially in prodromal HD cohorts, would be beneficial to validate the efficacy of this approach in detecting subtle changes in motor function.

In conclusion, the study highlights the potential of using accelerometer data and machine learning techniques for remote monitoring of upper limb function in individuals with HD and prodromal HD. The results suggest that this approach could serve as a valuable screening technique and aid in early intervention strategies for individuals at risk of developing HD, particularly when extended to larger sample sizes. Additionally, the ability to predict clinical scores, particularly the UHDRS UL, underscores the importance of remote monitoring in assessing disease progression and treatment response.

## Supplementary Material

Supplement 1

## Figures and Tables

**Table 1.** Participants Demographics and Clinical Characteristics (Mean ± Standard Error)

	HD (n=16)	pHD (n=7)	CTR (n=16)
Age, mean (SD)	51.9 (11.0)	36.5 (13.1)	58.9 (12.2)

Female, n (%)	8 (50.0)	6 (85.7)	11 (68.5)

Education level, n (%)			
Doctoral degree	1 (6.25)	0 (0.0)	0 (0.0)
Master’s degree	1 (6.25)	1 (14.2)	0 (0.0)
Some graduate school	0 (0.0)	0 (0.0)	2 (18.2)
Four-year college degree	5 (31.25)	0 (0.0)	3 (27.3)
Two-year college degree	2 (12.5)	1 (0.0)	4 (36.4)
Some college	1 (6.25)	2 (28.6)	1 (9.1)
High school diploma/GED	6 (37.5)	3 (42.9)	1 (9.1)

Race, n (%)			
American Indian or Alaska Native	0 (0.0)	1 (14.3)	0 (0.0)
White	16 (100)	6 (85.7)	14 (100)

Ethnicity, n (%)			
Not Hispanic or Latino	16 (100)	7 (100)	14 (100)

UHDRS			(n=11)
Total Functional Capacity, mean (SD)	8.8 (3.14)	12.5 (1.13)	12.7 (0.9)
Functional, mean (SD)	19.4 (4.5)	23.6 (1.1)	23.6 (1.2)
Motor, mean (SD)	45.7 (18.3)	1.7 (2.6)	0.7 (1.4)

CAG length, mean (SD)	45.7 (5.2)	44 (3.9)	

HD: Huntington’s disease, pHD: Prodromal Huntington’s disease, CTR: Controls, UHDRS: Unified Huntington’s Disease Rating Scale, SD: Standard Error

**Table 2.** Group means and statistical significance. All GDM metrics are averaged daily values measured over 7 consecutive days. The feature values are the daily averages. Bold statistics indicate significant differences, and * indicates surviving multiple comparison corrections.

	HD	pHD	CTR	HD vs pHD		HD vs CTR		pHD vs CTR	

	Mean ± std	Mean ± std	Mean ± std	Cohen’s D pval		Cohen’s D pval		Cohen’s D pval	
**Daily GDM counts**									
Daily GDMs, *n*	1110.6 ± 427.1	1046.6± 348	1217.36 ± 499.9	0.16	0.732	−0.23	0.521	0.37	0.428
Daily GDMs with duration > 4.5 s, *n*	805.92 ± 303.3	736.31 ± 226.5	796.04 ± 271.11	0.25	0.594	0.03	0.9235	0.23	0.616
Daily GDMs with duration > 7.5 s, *n*	98.98 ± 46.99	102.53 ± 51.76	286.9 ± 304.19	−0.07	0.873	**−0.86**	**0.0211** *	0.71	0.131
Daily GDMs with duration > 10.5 s, *n*	35.59 ± 18.87	37.46 ± 22.59	121.94 ± 136.17	−0.09	0.839	**−0.89**	**0.018** *	0.73	0.122
Daily GDMs with duration > 13.5 s, *n*	13.77 ± 7.95	15.28 ± 10.56	56.17 ± 66.85	−0.17	0.708	**−0.89**	**0.017** *	0.72	0.127
Daily GDMs with duration > 16.5 s, *n*	5.1 ± 2.99	5.96 ± 4.38	26.86 ± 34.4	−0.25	0.587	**−0.89**	**0.017** *	0.72	0.129

**Velocity features**									
Minimum velocity, *m/s*	18.46 ± 4.53	20.8 ± 3.11	19.21 ± 4.49	−0.56	0.230	−0.17	0.638	−0.38	0.409
Median velocity, *m/s*	59.03 ± 14.41	69.97 ± 7.85	69.77 ± 9.28	−0.85	0.075	**−0.89**	**0.0187** *	−0.02	0.961
Maximum velocity, *m/s*	109.95 ± 27.45	132.13 ± 11.3	132.09 ± 14.23	−0.93	0.054	**−1.01**	**0.008** *	0	0.995
Velocity root mean squared, *m/s*	67.42 ± 16.57	80.51 ± 8.19	80.4 ± 9.69	−0.89	0.062	**−0.96**	**0.011** *	−0.01	0.980
Entropy velocity	4.26 ± 0.01	4.26 ± 0	4.26 ± 0.01	0.45	0.337	0.18	0.619	0.04	0.929
Velocity zero crossings count, *n*	4.48 ± 0.68	4.53 ± 0.39	4.47 ± 0.39	−0.08	0.868	0.03	0.943	−0.16	0.734
Velocity zero crossings duration entropy	0.94 ± 0.03	0.96 ± 0.01	0.98 ± 0.02	**−0.98**	**0.043**	**−1.67**	**<0.001** *	**0.96**	**0.046**
Velocity zero crossings average duration, *n*	38.02 ± 2.58	39.25 ± 1.22	40.38 ± 2.94	−0.54	0.247	**−0.85**	**0.022** *	0.44	0.340

**Acceleration features**									
Minimum acceleration, *m/s*^*2*^	0.73 ± 0.23	0.92 ± 0.16	0.89 ± 0.18	−0.86	0.070	**−0.77**	**0.036**	−0.15	0.747
Median acceleration, *m/s*^*2*^	3.46 ± 1	4.1 ± 0.53	3.92 ± 0.64	−0.71	0.130	−0.55	0.129	−0.29	0.532
Maximum acceleration, *m/s*^*2*^	10.32 ± 2.98	11.09 ± 0.89	10.64 ± 1.38	−0.3	0.517	−0.14	0.704	−0.36	0.4363
Acceleration root mean squared, *m/s*^*2*^	4.37 ± 1.21	5 ± 0.51	4.86 ± 0.64	−0.6	0.198	−0.52	0.155	−0.23	0.615
Entropy acceleration	4.23 ± 0.02	4.24 ± 0	4.24 ± 0.04	−0.77	0.105	−0.37	0.299	0	0.999
Acceleration zero crossings count, *n*	35.47 ± 6.46	32.71 ± 3.43	31.07 ± 3.39	0.48	0.3023	**0.85**	**0.022** *	−0.48	0.298
Acceleration zero crossings duration entropy	1.04 ± 0.02	1.03 ± 0.01	1.02 ± 0.01	0.51	0.269	**0.99**	**0.008** *	−0.65	0.169
Acceleration zero crossings average duration, *n*	7.15 ± 1.49	7.88 ± 0.85	9.11 ± 2.36	−0.55	0.240	**−0.99**	**0.009** *	0.6	0.202

HD: Huntington’s disease, pHD: Prodromal Huntington’s disease, CTR: Controls, SD: Standard Error, pval: p-value, m: meters, s: seconds

**Table 3.** Model performance in predicting clinical scores.

	MSE	MAE	R	Explained variance
UHDRS Functional	11.5	2.4	0.45	0.18
UHDRS Motor	256.78	12.43	0.75	0.56
TFC	6.07	1.88	0.58	0.33
UHDRS UL	31.28	4.1	0.77	0.6

MSE: mean squared error, MAE: mean absolute error, R: correlation score, TFC: Total functional capacity, UL: upper limb
