# Supplementary material for: Using Wearable Sensors and Machine Learning to Assess Upper Limb Function in Huntington’s Disease
Source: Res Sq. 2024 Jun 3:rs.3.rs-4355136. Preprint. [Version 1] doi: 10.21203/rs.3.rs-4355136/v1 (PMC11177990; doi:10.21203/rs.3.rs-4355136/v1)
Supplement: Supplement 1 [file NIHPPrs4355136v1-supplement-1.pdf]

Supplementary material

Table S1. GDM features correlations with clinical assessment scores. All GDM metrics are averaged daily values measured over 7 consecutive days. Bold statistics indicate significant differences, and * indicates surviving multiple comparison corrections.

	**UHDRS Functional**		**UHDRS Motor**			**TFC**			**UHDRS UL**		
	**corr**	**pval**	**corr**	**pval**		**corr**	**pval**		**corr**	**pval**	
**GDM counts features**											
GDM, *n*	0.09	0.607	0.12	0.510		0.00	0.999		0.02	0.923	
GDM with a duration < 4.5 s, *n*	0.02	0.921	0.20	0.280		-0.08	0.640		0.10	0.587	
GDM with a duration > 7.5 s, *n*	0.20	0.264	-0.01	0.977		0.14	0.432		-0.07	0.681	
GDM with a duration > 10.5 s, *n*	0.22	0.213	-0.04	0.817		0.16	0.369		-0.11	0.520	
GDM with a duration > 13.5 s, *n*	0.24	0.171	-0.04	0.819		0.18	0.300		-0.10	0.565	
GDM with a duration > 16.5 s, *n*	0.20	0.266	0.00	0.998		0.15	0.402		-0.06	0.741	
**Velocity features**											
Minimum velocity, *m/s*	0.29	0.093	-0.34	0.058		0.28	0.108		**-0.34**	**0.046**	
Median velocity, *m/s*	**0.39**	**0.021**	**-0.50**	**0.004**	*	**0.41**	**0.016**		**-0.52**	**0.002**	*
Maximum velocity, *m/s*	**0.46**	**0.006**	**-0.53**	**0.002**	*	**0.47**	**0.005**	*	**-0.56**	**0.001**	*
Velocity root mean squared, *m/s*	**0.42**	**0.015**	**-0.51**	**0.003**		**0.44**	**0.010**	*	**-0.53**	**0.001**	*
Entropy velocity	-0.21	0.225	0.26	0.155		-0.29	0.102		0.30	0.083	
Velocity zero crossings count, *n*	0.03	0.880	-0.07	0.694		0.04	0.813		-0.02	0.921	
Velocity zero crossings duration entropy	**0.48**	**0.004**	**-0.71**	**0.000**	*	**0.59**	**0.000**	*	**-0.67**	**0.000**	*
Velocity zero crossings average duration, *n*	0.21	0.236	-0.32	0.071		0.27	0.124		-0.30	0.082	
**Acceleration features**											
Minimum acceleration, m/s^2	**0.43**	0.012	-0.51	0.003	*	0.44	0.009	*	**-0.54**	**0.001**	*
Median acceleration, m/s^2	**0.35**	0.044	-0.39	0.027		0.36	0.040		**-0.42**	**0.014**	*
Maximum acceleration, m/s^2	0.15	0.406	-0.19	0.296		0.10	0.566		-0.16	0.362	
Acceleration root mean squared, m/s^2	0.30	0.088	**-0.36**	**0.043**		0.29	0.093		**-0.36**	**0.035**	
Entropy acceleration	**0.35**	**0.042**	**-0.50**	**0.003**	*	**0.41**	**0.016**		**-0.46**	**0.006**	*
Acceleration zero crossings count, *n*	-0.31	0.073	0.31	0.082		**-0.37**	**0.030**		**0.40**	**0.020**	
Acceleration zero crossings duration entropy	-0.28	0.116	0.35	0.050		**-0.34**	**0.049**		**0.38**	**0.026**	
Acceleration zero crossings average duration, *n*	**0.42**	**0.013**	**-0.47**	**0.007**	*	**0.51**	**0.002**	*	**-0.52**	**0.001**	*

TFC: Total functional capacity, UL: upper limb, pval: p-value, m: meters, s: seconds, corr: correlation
